# Involvement of Netrin/Unc-5 Interaction in Ciliary Beating and in Pattern Formation of the Ciliary Band-Associated Strand (CBAS) in the Sea Urchin, *Hemicentrotus pulcherrimus*

**DOI:** 10.3390/ijms21186587

**Published:** 2020-09-09

**Authors:** Hideki Katow, Kouki Abe, Tomoko Katow, Hiromi Yoshida, Masato Kiyomoto

**Affiliations:** 1 Research Center for Marine Biology, Tohoku University, Asamushi, Aomori 039-3501, Japan; k-abe@bs.naist.jp (K.A.); h.tkatow@d6.dion.ne.jp (T.K.); 2 Institute of Development, Aging and Cancer, Tohoku University, Sendai 980-8575, Japan; hiromi.yoshida.a6@tohoku.ac.jp (H.Y.); kiyomoto.masato@ocha.ac.jp (M.K.); 3 Marine and Coastal Research Center, Ochanomizu University, Tateyama, Chiba 294-0301, Japan

**Keywords:** CBAS, pattern formation, Netrin/Unc-5 interaction, 3D reconstruction, sea urchin larva

## Abstract

The GABAergic neural circuit is involved in the motile activities of both larval and juvenile sea urchins. Therefore, its function is inherited beyond metamorphosis, despite large scale remodeling of larval organs during that period. However, the initial neural circuit formation mechanism is not well understood, including how glutamate decarboxylase-expressing blastocoelar cells (GADCs) construct the neural circuit along the circumoral ciliary band (a ciliary band-associated strand, CBAS) on the larval body surface. In this study, using whole-mount immunohistochemistry and 3D reconstructed imaging, the ontogenic process of CBAS patterning was studied by focusing on Netrin and the interaction with its receptor, Unc-5. During the early 2-arm pluteus stage, a small number of GADCs egress onto the apical surface of the larval ectoderm. Then, they line up on the circumoral side of the ciliary band, and by being inserted by a further number of GADCs, form longer multicellular strands along the Netrin stripe. Application of a synthetic peptide, CRFNMELYKLSGRKSGGVC of Hp-Netrin, that binds to the immunoglobulin domain of Unc-5 during the prism stage, causes stunted CBAS formation due to inhibition of GADC egression. This also results in reduced ciliary beating. Thus, the Netrin/Unc-5 interaction is involved in the construction and function of the CBAS.

## 1. Introduction

The swimming activity of sea urchin embryos is initially a spinning movement on the animal–vegetal axis in the fertilization envelope. After hatching, the embryos swim with the animal pole forward and rotate counterclockwise on the animal–vegetal axis, and then change to a transient reversal swimming direction at the pluteus stage [1]. At and after the swimming blastula stage, glutamate decarboxylase transcript accumulation in *Hemicentrotus pulcherrimus* (Hp-GAD), a γ-aminobutyric acid (GABA) synthetase, is particularly prominent in clumps of ectodermal cells throughout the embryonic surface. During the gastrula stage, the transcripts also accumulate in the endomesodermal cells and certain blastocoelar cells. During further development, Hp-GAD transcripts accumulate in the ectodermal cells and then in the blastocoelar cells, along with the appearance of GABA-immunopositivity in these cells (GADC) [2]. The subpopulation of these GADCs egress to the larval surface near the ciliary band during the 2-day post-fertilization 2-arm pluteus stage (2dpf-2aPL) [3]. 

From the 2aPL stage, swimming activity increases, which is associated with the occurrence of ectodermal cells that accumulate Hp-GAD transcripts, along with its proteins’ immuno-positive signals on the apical side of the ciliary band [3]. During a further developmental period, from the 4aPL to 6aPL stage, GADCs line up between the circumoral and aboral ectodermal regions along the ciliary band, forming the ciliary band-associated strand (CBAS) by expressing serotonin receptor (5HThpr), encephalopsin (ECPN), synaptophysin (Syn), and GABA (A) receptor [3,4,5]. The larval swimming speed increases during these pluteus stages, with more complex swimming patterns [6] in order to facilitate feeding c.f. [7]. 

After metamorphosis, the primary podia express GAD, along with GABA(A) receptor, and mobility is severely inhibited under the presence of 3-mercaptopropionic acid [8]. Consistent with the above presence of the GABA signaling cascade in the tube feet of the adult, GABA involvement in tube feet movement in adult sea urchins has been reported as an excitatory neurotransmitter that functions through excitation of cholinergic motoneurons. Accordingly, GABA has been isolated from the tube feet musculature [9]. More specifically, this neurotransmitter was detected at the ectoderm neuromuscular junction, along with GABA (A) and (B) receptor subtypes [10]. 

Thus, the GABAergic signal transmission system (GSTS) consistently plays a major role in sea urchin mobile behavior from the ciliary band, in plutei to primary podia after metamorphosis [8]. However, motility of sea urchin larvae is not solely regulated by the GSTS, but also by the serotonergic system or synaptotagmin (Synt)-expressing neural system [11]. On the basal side of the ciliary band ectoderm, the Synt-expressing neuronal fibers encircle the circumoral ectoderm, and its apical ganglion region colocalizes with the serotonergic ganglion in sea urchin larvae [12]. The basal surface of the serotonergic apical ganglion bilaterally extends short axons [13] that express Hp-Unc-5, and whose extension is depleted by Hp-Unc-5 morpholino [14]. Bilateral extension of these axons is interrupted by the presence of a synthetic peptide SQDFGKTW that constitutes the Netrin’s binding site to Unc-5 [14,15], implicating the fundamental role of the Netrin/Unc-5 interaction in axonal extension in sea urchin larvae.

In this study, the involvement of the Netrin/Unc-5 interaction in CBAS formation was examined using (1) whole-mount immunohistochemistry, which was further analyzed with (2) 3D-reconstructed images, (3) by intervention with Netrin peptide. These observations were further analyzed by (4) bioassay of the larval swimming behavior. Finally, to estimate the potential continuity of molecular components among the CBAS forming cells, their cytoplasmic properties were examined by immunohistochemistry.

## 2. Results

### 2.1. Blastocoelar GAD-Expressing Cell Egression onto the Apical Surface of Larval Arms and Lining Up for CBAS Patterning

**Figure 1:**
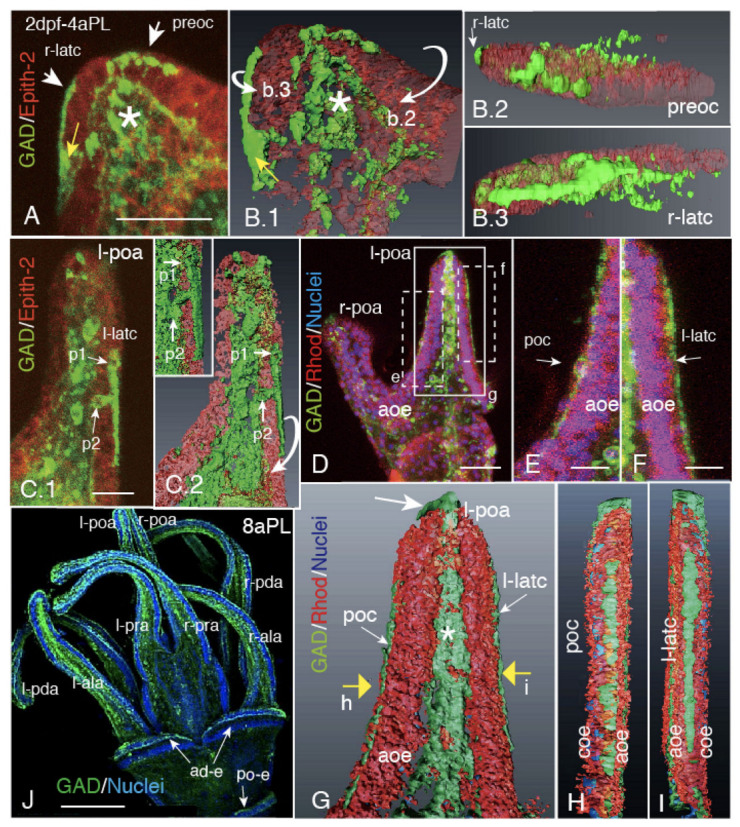
CBAS patterning process during the 4aPL stage. (**A**) Confocal laser scanning microscopy (CLSM) of the right antero-lateral arm showing a mass of blastocoelar GAD-positive cells (GADC, asterisk) egressed in part onto the larval body surface at the arm tip (arrows). (*B.1) 3D-reconstructed image of (A). Part of the egressed GADCs extend as a fragmental stripe of preoral CBAS (preoc) on the apical surface of the antero-lateral arm tip. The other GADCs are egressed onto the other direction and extended to form a short preoral CBAS (preoc). (**B.2**) Rotated image of (B.1) toward the direction shown by a curved arrow, b.2, in (B.1), showing the fragmental stripe of preoral CBAS. Part of the right lateral CBAS is seen (r-latc). (**B.3**) Rotated image of (B.1) as shown by the curbed arrow b.3 in (B.1). GADCs extend as a stripe on the larval arm surface. (**C.1**) CLSM image of the left post-oral arm. A posteriorly extended fragmental CBAS (l-latc) retains perikarya on the apical surface (p1) and basally in the ectoderm (p2). (*C.2) 3D-reconstructed image of (C.1). (C.2, inset) Rotated image of (C.2) as shown by a curbed arrow. (**D**) CLSM of the oral side of the larva shows the left- and right-post-oral arm region (l-poa, r-poa). Two CBASs are seen between the aboral ectoderm (aoe) and the circumoral ectoderm (coe). (**E**) Higher magnification of the post-oral CBAS (poc) with perikaryon, shown by a rectangle (e) in (D). (**F**) Higher magnification of the left lateral CBAS (l-latc), shown by a rectangle (f) in (D). (*G) Aboral view of 3D-reconstructed image of the same area, as shown by a rectangle (g) in (D). The poc and the l-latc are separated from the blastocoelar GADCs (asterisk), while some blastocoelar GADCs are egressed at the tip of the arm (arrow). (**H**) The poc in (G), viewed from the direction shown by a yellow arrow (h) in (G). (**I**) The l-latc shown in (G), viewed from the direction shown by a yellow arrow (i) in (G). (**J**) Fully formed circumoral CBAS and those on the oral side antero-dorsal-epauletes (ad-e) and posterior-epaulet (po-e). * Refer to Supplemental Figure S1(B.1,C.2,G). Scale bars = 25 μm (A, C.1), 10 μm (D–F), 100 μm (J).

Masses of blastocoelar GADCs egressed from several places on the larval arms to their apical surface. In 2dpf-early 4aPL, the GADCs egressed in the vicinity of the tip region of the antero-lateral arm (ala) (Figure 1A). These GADCs retained their perikaryon in the ectoderm (Figure 1(A,B.1), yellow arrow), or extended a part of their cell body by leaving the perikaryon region in the blastocoel that held a GADC mass with other blastocoelar GADCs (Figure 1(A,B.1), asterisk). A part of these GADCs elongated linearly on the apical surface by forming preoral CBAS (Figure 1B.2, preoc) and lateral CBAS (Figure 1(A,B.1,B.3), r-latc). Some of these initial CBASs that egressed at the middle of the post-oral arm were composed of two cells due to leaving a cell within the ectoderm, such as shown by p1 and p2 in Figure 1(C.1,C.2).

These CBASs, along with the entire cell body, extended further on both sides of the post-oral arm (Figure 1D–F). According to the 3D-reconstructed images of the same regions as above (Figure 1G), we detected that these two CBASs, the post-oral CBAS (poc) (Figure 1H) and the left-latc (Figure 1I), did not spread broadly on the apical surface, but aligned by forming a narrow strand between the circumoral (coe) and aboral ectoderms (aoc). By the 8aPL stage, all eight larval arms were divided into the circumoral ectoderm and the aboral ectoderm regions by CBAS (Figure 1J). During this developmental stage, a process for metamorphosis starts that causes dissolution of the larval CBAS [8]. Two isolated CBASs that are associated with the antero-dorsal epaulets (Figure 1J ad-e), and one associated with the posterior epaulet at the most posterior region (Figure 1J, po-e), were derived from the circumoral CBAS between the 6aPL and 8aPL stage [16].

**Figure 2:**
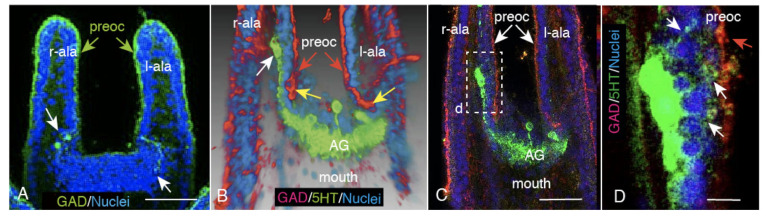
Circumoral CBAS interruption at the oral side of the upper lip field of 4aPL. (**A**) Confocal laser scanning microscopy (CLSM) of preoral CBAS (preoc) shows suspension at the upper lip field (white arrows). (*B) 3D-reconstructed image of (C) shows the oral side view around the serotonergic apical ganglion (AG, green). The terminal ends of the left- and right-preocs are shown with yellow arrows. White arrow; one of the bilateral arms from the apical ganglion. (**C**) CLSM of (B). The serotonergic apical ganglion (AG) extends an axon toward the right antero-lateral arm (r-ala) beneath the ectoderm. (**D**) Higher magnification of the rectangle (d) in (C) shows several cell processes from the perikaryon reaching to the preoral CBAS (white arrows). Red arrow: preoral CBAS. Scale bars = 30 μm (A), 25 μm (C), 5 μm (D). * Refer to Supplemental Figure S2B.

Although the CBAS almost completely encircled the circumoral ectoderm by the end of 4aPL, it was not perfect encircling. There was a discontinued site by a large gap around the apical ganglion in the upper labial part (Figure 2A, allows). The gap area housed the serotonergic apical ganglion that extended bilateral branches towards the basal side of the antero-lateral arm ectoderm (Figure 2B, arrows). A terminal perikaryon of the branch in the antero-lateral arms that were extended anteriorly from the apical ganglion (Figure 2B, white arrow) extended several thin and long cell processes toward the CBAS (Figure 2C, rectangle (d); Figure 2D, white arrows). Thus, the circumoral CBAS was divided by the left and right hemi-circuits near the apical ganglion, and the ganglion itself seemed to mediate between these hemi-circuits.

**Figure 3:**
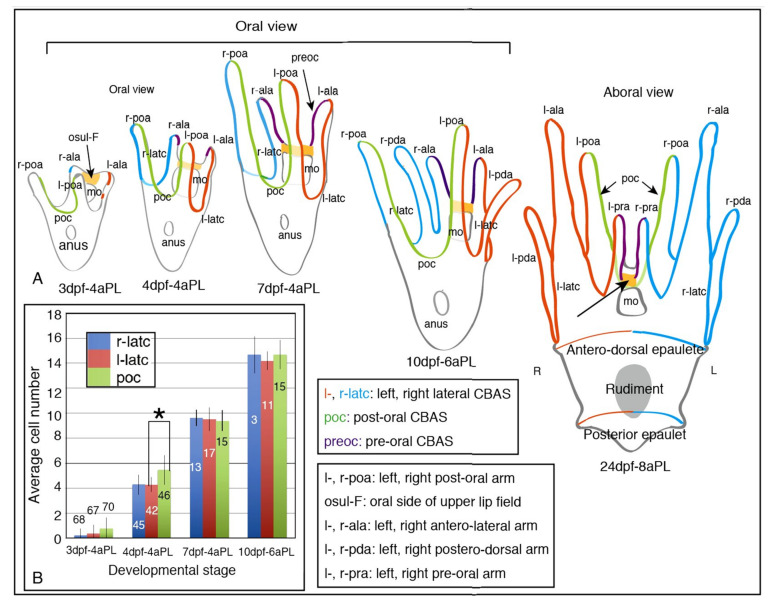
Schematic summary of the CBAS pattern formation. (**A**) Schematic process of the CBAS pattern formation from the 3 days post-fertilization, 4-arm pluteus stage (3dpf-4aPL) to 24dpf-8aPL, immediately before metamorphosis. The long arrow on 24dpf 8aPL: suspended CBAS near the apical ganglion. (**B**) Increasing cell number of the CBAS from 3dpf-4aPL to 10dpf-6aPL, with error bars. Asterisk: the number of post-oral CBAS (poc)-constituting cells is statistically significantly larger than that of r- and l-latc. *p* = 0.0001 < 0.05. The number on each column is the total number of larvae subjected to count the cell number in the CBAS.

The above CBAS patterning process was summarized by a schematic (Figure 3A). Extension of these CBAS was associated with an increasing number of strand-composing cells, that were led by poc forming cells from the 3d-4aPL stage to the 4dpf-4aPL stage (Figure 3B, asterisk). Those of the left and right lateral CBAS were almost synchronized. However, the eventual cell number of poc and l- and r-latc at and after the 7dpf-4aPL stage were almost equal, at least by the 10dpf-6aPL stage (Figure 3B).

Although all of the above CBASs, except those at the posterior and the antero-dorsal epaulets, appeared to be connected as a single strand, they were clearly interrupted around the serotonergic apical ganglion (Figure 2B). The closest antero-lateral CBAS was also not connected with the ganglion, implicating the incomplete circumoral connection of the CBAS patterning.

### 2.2. Heterogeneous Protein Expression among CBAS Component Cells

**Figure 4:**
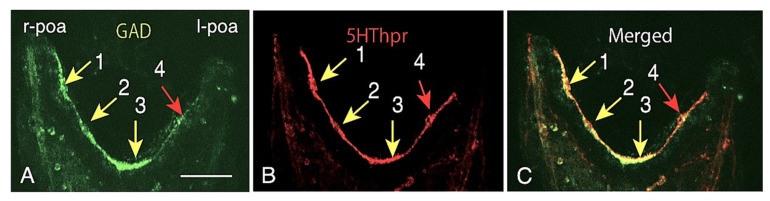
Heterogeneous expression intensities of cytoplasmic proteins among the post-oral-arm CBAS forming cells in 4aPL. (**A**) The intensity of GAD expression among the four cells is different; #3 cell expressed the most intensively, while #4 cell was the weakest, with the other two cells in between. (**B**) 5HThpr was expressed the most intensively in the #1 and #3 cells, while the other two cells were weaker. (**C**) Merged image between (A) and (B). Scale bar = 30 μm.

The circumoral CBAS formation did not take place with simultaneous construction of its continuity as a strand; it was initially fragmental forms (Figure 1). This raised the question of how these initial inter-GADCs interactions take place. Does the cytoplasm retain individuality? To answer this question, two CBAS proteins, GAD and 5HThpr [4], were examined immunohistochemically. Double-stained WMIHC using anti-GAD Ab and anti-5HThpr Ab of the poc of 4aPL indicated that these two proteins had different expression intensities among the cells (Figure 4). GADC-3, -1, -2 cells showed the strongest intensity of GAD expression, while GADC-4 cells showed the weakest (Figure 4A). 5HThpr-1, -3, -2 cells showed the strongest 5HThpr expression, while 5HThpr-4 cells showed the weakest (Figure 4B). Therefore, among all four GADCs, the intensity of the GAD-positive signal and that of the 5HThpr signal was quite different (Figure 4C). That in the proteins of GAD and 5Hthpr their expression intensity was different from cell to cell suggested that these CBAS composing cells retain their individuality. This also implied that there is little or no cytoplasmic continuity during the pattern formation stages. 

### 2.3. CBAS Locates between the Netrin Stripe on the Aboral Side and the Netrin-Free Oral Side of the Circumoral Ectoderm 

**Figure 5:**
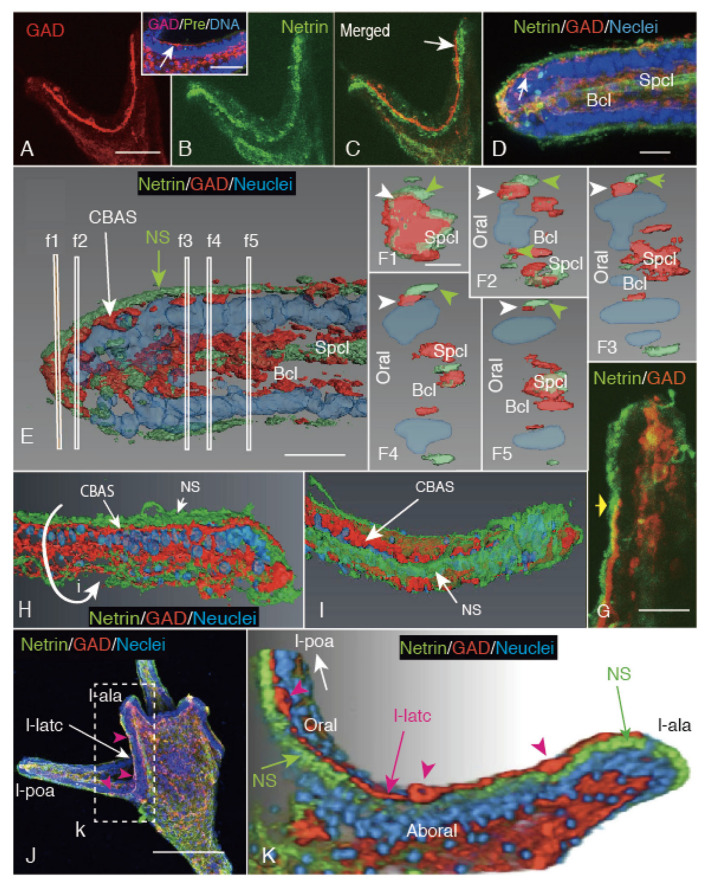
Pattern formation of GAD-positive CBAS (red) along the Netrin-positive stripe in 4aPL. (**A**) Confocal laser scanning microcopy (CLSM) of the post-oral CBAS (poc). (**B**) The Netrin stripe along the poc. (**C**) Merged image between (A) and (B). Inset: pre-immune serum of anti-Netrin Ab applied poc (arrow) area. (**D**) CLSM of a post-oral arm region. Netrin (green) layer was on the apical surface and on the spicule surface (Spcl) in the blastocoel (Bcl). GAD-positive CBAS (red) is seen beneath the Netrin-positive layer on the apical surface (arrow). (**E**) 3D-reconstructed image of (D). Thin vertical rectangles locate the following cross-sectional images of the area shown by (F1) to (F5). (**F1**–**F5**) These cross sections consistently located the CBAS (white arrowheads) on the circumoral ectoderm side of the Netrin strip (green arrowheads), as is seen with the flat oval cut-surface. (**G**) CLSM of a longitudinal center line section of a post-oral arm used for 3D-reconstruction images (H) and (I). (**H**) The CBAS (red) is seen beneath the Netrin-positive layer (NS, green). (**I**) 3D image of (G) that was tilted along the curved arrow in (H) showing the arm surface from the direction pointed by a yellow arrow in (G), indicating the Netrin stripe (NS) on the CBAS. (**J**) CLSM of the whole-body of the aboral side of a larva, and the left-lateral CBAS (l-latc) extends from the left antero-lateral arm (l-ala) to the left post-oral arm (l-poa). (*K) 3D image of (J) shown by a rectangle (k) in (D) locates l-latc (red) between the Netrin stripe (NS) and circumoral ectoderm area (Oral). Scale Bars = 25 μm (A), 10 μm (D, E, F1), 25 μm (G). * Refer to Supplemental Figure S5K.

The characteristic stripe pattern of GADCs after egression onto the apical surface of the larval arms (Figure 1 and Figure 3) suggest the presence of a GADC patterning navigation cue, possibly by an extracellular matrix (ECM). Netrin has been reported in larval serotonergic nervous system formation in sea urchins [14,17]. To elucidate the presence of similar ECM localizations on the apical surface of the larval arms, 4aPL was double-stained with Hp-GAD Ab for the CBAS [2,4] and Hp-Netrin Ab for the Netrin stripe [17]. Immunohistochemically, the poc (Figure 5A) was closely co-localized with the narrow part of the Netrin-positive stripe (Figure 5B). The merged image indicated a consistent close distribution between these two signals, with a slightly larger area of the Netrin stripe than that of CBAS (Figure 5C). More specifically, the merged image located the poc on the circumoral ectoderm side of the Netrin stripe (Figure 5C, arrow). Further spatial analysis was conducted using the above CLSM of a triple-stained post-oral arm. This was located the Netrin-positive area on the very outer surface of the ectoderm, while the CBAS was localized beneath the Netrin layer (Figure 5D). Some blastocoelar components and spicule surfaces were also Netrin-positive. The 3D-reconstructed image of the above CLSM image clearly located the Netrin stripe on the apical surface of the GAD-positive CBAS, and the Netrin-positive ECM around some GAD-positive blastocoealar cells (Figure 5E). This implied possible multiple functions of the Netrin-positive extracellular matrix. The topological relationship between the Netrin stripe and the CBAS was analyzed by a series of digital optical cross-sections made at several places, as shown by slim rectangles (Figure 5. f1 to f5). Throughout these optical cross-sections, the Netrin-positive areas presented flattened oval shapes on the outer surface, while the CBAS was consistently localized on the circumoral ectoderm side beneath the Netrin stripe. This indicated that the CBAS is located on the circumoral ectoderm side, and diagonally below the Netrin stripe. At the tip region of the arm, however, the CBAS occupied a large cross-sectional area (Figure 5(E,F1)), indicating the area was where the blastocoelar GADCs are egressing. The abovementioned spatial distribution pattern of the Netrin stripe and CBAS was further analyzed using other 3D-reconstructed images, made based on a CLSM image (Figure 5G). The 3D image clearly indicated that the Netrin stripe on the apical surface of the CBAS was positioned on the circumoral ectoderm side of the Netrin stripe (Figure 5H,I). 

The lateral CBAS of 4aPL included several perikarya (Figure 5J, red arrowheads), and the 3D-reconstructed image of the area depicted clear localization of the CBAS in between the Netrin-negative circumoral ectoderm and the Netrin stripe on the aboral ectoderm side (Figure 5K).

### 2.4. Netrin Stripe along the Ciliary Band

**Figure 6:**
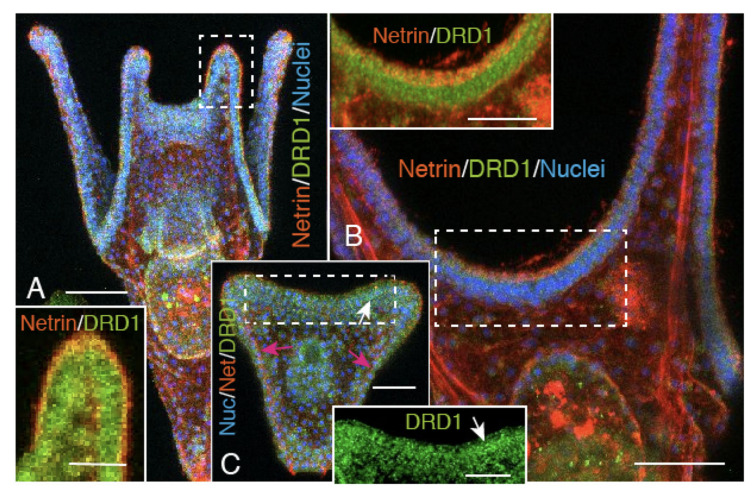
Confocal laser scanning microscopy of the Netrin stripe formed on the oral side of the DRD1-expressing ciliary band in 4aPL, and lack of the Netrin stripe in very early 2aPL. (**A**) Aboral side view. A Netrin-positive signal is detected on the apical side of the DRD1-expressing circumoral ciliary band. Inset: higher magnification of the right antero-lateral arm shown by a rectangle in (A). (**B**) Oral side of the post-oral CBAS. Inset: Netrin/DRD1 stained image of the rectangle in (B) shows a partially overlapped Netrin stripe and DRD1-positive ciliary band. Inset: Netrin stripe on the circumoral ectoderm side of the DRD1-positive ciliary band. (*C) Close to the end of the prism stage. The DRD1-positive ciliary band is not associated with the Netrin stripe but remained in the blastocoel (red arrows) in this developmental stage. Inset: higher magnification of the ciliary band region (arrow) shown by the rectangle in (C). Scale bars = 50 μm (A), 25 μm (insets of A and C), 30 μm (B, C), 15 μm (inset of B).

The above observations strongly resembled the spatial relationship between the CBAS and the dopamine receptor D1 (DRD1)-expressing ciliary band reported previously [18]. To examine the occurrence of such a spatial relationship, and to locate the CBAS position precisely, it was examined by WMIHC using anti-sea urchin DRD1 antibody [18,19]. The triple stained WMIHC detected the Netrin stripe on the circumoral ectoderm along the DRD1-positive ciliary band on the larval arms of 4aPL (Figure 6A and inset, B and inset). At the period between near the end of the prism stage and the early 2aPL stage, despite the clear presence of the DRD1-positive ciliary band between the left and right antero-lateral arms, no Netrin stripe was detected along the ciliary band, and some only in the blastocoel (Figure 6C and inset, red arrows), indicating the circumoral ciliary band is already formed before the appearance of the Netrin stripe. This temporal relationship with the ciliary band formation is consistent with a previous report by Giudice [20], and implicates that the Netrin gene is transcribed around the 15 hpf swimming blastula stage [17]. A close spatial association of the Netrin stripe with the ciliary band on the larval surface occurred later, by the 2dpf 4aPL stage (Figure 6A,B). The spatial closeness between the Netrin stripe and the DRD1-expressing ciliary band may occur near the early 2aPL stage, and this appears to be in good agreement with Lv-Netrin in situ reports on the circumoral ectoderm along the ciliary band by Slota et al. [21]; this will be further discussed later.

### 2.5. Unc-5 Expression in the CBAS on the Oral Side of the Netrin Stripe and Involvement of the Netrin/Unc-5 Interaction in Larval Ciliary Beating and CBAS Pattern Formation

**Figure 7:**
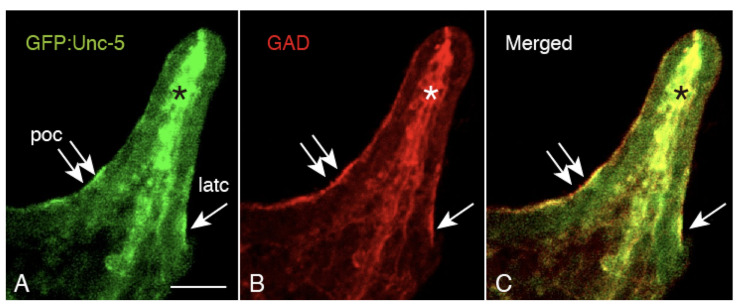
Confocal laser scanning microscopy of 4dpf-4aPL shows co-expression of Unc-5 and GAD at the CBASs. (**A**) GFP:Unc-5-positive post-oral CBAS (poc) and lateral CBAS (latc). Asterisk: GFP:Unc-5-positive cells in the blastocoel. (**B**) GAD-positive, same area as (A). Inset: GAD-positive cells in the blastocoel. (**C**) Merged image between (A) and (B). Double arrows: poc; single arrow: lateral CBAS. Scale bar = 30 μm.

**Figure 8:**
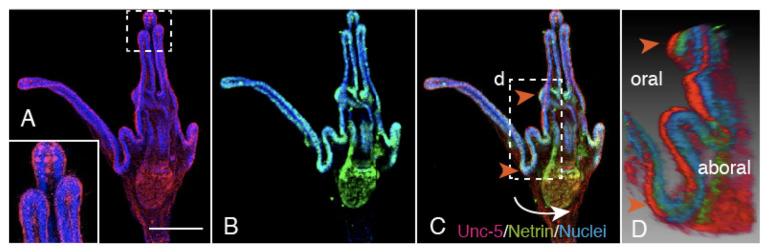
Aboral view of triple-stained 10dpf-6aPL with Unc-5 (red), Netrin (green), and Nuclei (blue). (**A**) Confocal laser scanning microscopy (CLSM) shows the Unc-5-positive CBAS. Inset: higher magnification of the rectangle shows Unc-5-positive cells in the blastocoel of the arm tip. (**B**) CLSM of the same larva shows the Netrin stripe (green). (**C**) Merged image between (A) and (B) shows the CBAS on the Netrin stripe. (*D) 3D-reconstructed image of the area, indicated by a rectangle in (C), was rotated to the direction shown by a curved arrow to show the Unc-5-positive CBAS on the oral side of the Netrin stripe (red arrowheads in C and D). Scale bar = 75 μm. * Refer to Supplemental Figure S8D.

To locate the Netrin receptor, the presence of Unc-5, one of its receptors, was examined using GFP-tagged Hp-Unc-5v2 [14]. GFP:Unc-5 was detected at the poc in 4aPL (Figure 7A, double-arrows). The positive signal was colocalized with an anti-GAD positive signal (Figure 7B,C, double-arrows), implying that the CBAS expresses the Netrin receptor. Accordingly, the present WMIHC detected that the Unc-5-positive CBAS was localized on the oral side of the Netrin stripe on the larval arms (Figure 8). Since the Unc-5/Netrin interaction contributes to serotonergic axon extension and swimming activity of the larvae [14], it was predicted that if such a receptor/ligand interaction is also involved in the CBAS and Netrin stripe, the interaction may also contribute to ciliary beating as well. Thus, we applied a peptide that was synthesized by referring to the Unc-5 interaction site of Netrin, CRFNMELYKLSGRKSGGVC [22]. This sequence is also found in Hp-Netrin, as described in Section 4.

**Figure 9:**
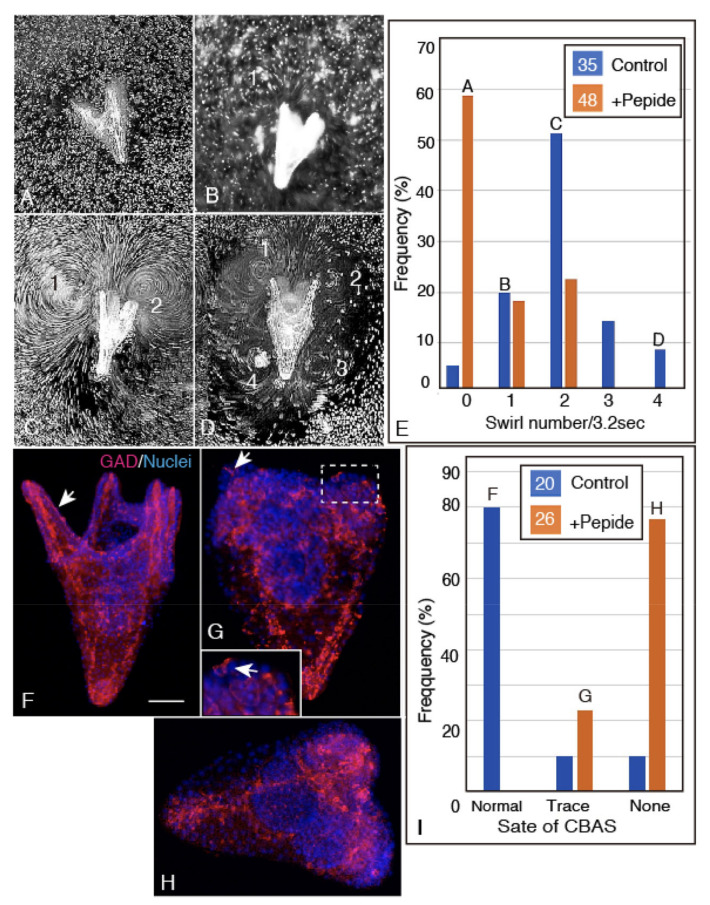
Application of Netrin peptide interferes with ciliary beating activity and CBAS patterning in 3dpf 4aPL. (**A**) A major proportion of the peptide-applied larvae formed no swirl (Column A in (E)). (**B**) A second major proportion of the control larvae formed one swirl (Column B in (E)). (**C**) A major proportion of control (Column C in (E)) and a distant second major proportion of peptide-applied larvae formed two swirls. (**D**) A small proportion of control larvae formed four swirls (Column-D in (E)). (**E**) The frequency of swirls in terms of group distribution was examined in 35 control larvae (blue columns) and 22 peptide-applied larvae (brown columns). Letters on the top of each column represent swirl patterns A–D. (F–I) Fluorescence microscopy of the whole-mount immunohistochemistry of 4aPL. (**F**) The major CBAS pattern of the control 4aPL acquired the clear poc (arrow). (**G**) A small proportion of the peptide-applied 4aPL formed CBAS composed only of a few GADCs insertions into the circumoral ectoderm (arrows, rectangle). Inset: enlarged rectangle area. (**H**) No CBAS was formed in the majority of peptide-applied larva. (**I**) The frequency of CBAS forms in the control larvae (blue columns, total 20 larvae) and in the peptide-applied larvae (brown columns, total 26 larvae). Letters on the top of columns represent the CBAS forms shown by the WMIHC images (F–H). Scale bar = 60 μm.

The ciliary beating of 4aPL was visualized as swirling track patterns obtained under the presence of sea algae as a marker for dark-field overexposed microscopy, as has been reported before [23]. The present examination detected that, in a total of 22 peptide-treated larvae, 29% produced two swirls, the rest (18.8%) produced one or none at all (52%) (Figure 9A,E). On the other hand, in a total of 35 control larvae, 84.2% formed two to four swirls (Figure 9B–D), the rest (20%) formed one or (5.7%) none (Figure 9A,E). Thus, the Netrin/Unc-5 interaction may contribute to the beating activity of the ciliary band.

To verify this, the CBAS pattern formation was immunohistochemically examined as follows. At the 4aPL stage, the control larvae formed the normal poc (Figure 9F, arrow), while in the peptide-applied larvae, the CBAS remained in trace (arrow in Figure 9G and inset) or totally deprived (Figure 9H). The frequency of each type of CBAS formation in a total of 46 larvae was as follows; in 20 control larvae, 80% of them formed normal poc, 10% formed CBAS composed of one to two GADCs, and the last 10% formed no CBAS at all; in 26 peptide-applied larvae, 77% formed no CBAS at all, and 23% formed a trace CBAS (Figure 9I). Therefore, the occurrence of deteriorated CBAS patterning and of reduced ciliary beating activity to produce swirls were well-correlated consequences of peptide application.

In addition to a disturbance to the CBAS patterning, peptide application also resulted in shorter arms with poor spicule extension, with a poor association of blastocoelar GADCs along the long axis of the arms. Instead, these GADCs accumulated on the basal side of the anterior ectoderm (Figure 9G,H).

## 3. Discussion

### 3.1. Blastocoelar GADC Egression onto the Apical Surface of the Larval Arms and Lining Up for the CBAS Patterning 

One of the initial crucial morphogenetic movements in sea urchin embryogenesis is the primary mesenchyme cell ingression that occurs immediately before the onset of gastrulation, which is then followed by various types of mesenchymal cells from the single layered ectoderm establishing three germ layers by the gastrula stage [20,24]. Thus, there are several occasions in which the ectoderm sheds migratory cells by ingression into the blastocoel during embryogenesis. However, no such migratory cell production through the egression onto the embryonic apical surface has been reported to date in sea urchin development. Egression of migratory cells, however, has been frequently reported in the later stage of development in neurogenesis in numerous animals, such as hematopoietic stem cell (HPSC) egression from the bone marrow during sympathetic nervous system development in mice [25], and the first-generation neuronal precursors in the brain of crayfish, *Procambarus clarkii* [26]. These migratory cell egressions occur in response to exogenous factors, such as granulocyte colony-stimulating factor for HPSCs [25], and the extracellular matrix (ECM) and basal lamina in the crustacean brain [26]. Even in these latter cases, however, the egression occurs inside the body cavity but not onto the outer surface of the body, which is similar to the mesenchyme cell ingression in the sea urchin embryo. The common environment would be the presence of the ECM, such as the basal lamina in sea urchin embryos. Thus, ECM signaling is one of the key elements regulating egression, as will be discussed later.

The first event of blastocoelar GADC egression is, as the primary mesenchyme cell ingression, to cross the basal lamina on the basal surface of the ectoderm and then break the intercellular junctions near the apical surface, such as the septate desmosomes between the ectodermal cells [27,28]. In addition, the present egression is followed by the establishment of new intercellular junctions between these egressed GADCs, to form the circumoral CBAS on the larval body surface. These intercellular junctions are the Gap junction-type connections that transmit small molecules, such as protein molecules which are less than 1kDa in size [29]; GAD as was shown as an example of this in the present study (Figure 4). Hp-Gad attains a size of about 60.137 kDa without sugar chains (http://cell-innovation.nig.ac.jp/cgi-bin/Hpul_public/Hpul_annot_search_output.cgi). Thus, it is too large to pass through the Gap junctions. Although the Gap junction constituents, connexin and their genes, are detected in the sea urchin genomic database of *H. pulcherrimus* [30] (HpBase; http://cell-innovation.nig.ac.jp/cgi-bin/Hpul_public/Hpul_annot_search_output.cgi), to date, neither connexin nor pannexin has been detected by an HMMER: a biosequence analysis using profile hidden Markov models [31]. Although the presence of functional and structural Gap junctions still needs to be further examined [31,32], it could be conceivable that Gap junction-type connections in the CBAS divide the cytoplasmic proteins, such as GAD, in adjacent cells, preventing them from crossing the intercellular boundary (Figure 4). Regarding the EchinoBase reports suggesting the presence of KIAA1432 protein-like and connexin 43-interacting protein 150 (http://legacy.echinobase.org/Echinobase/), Gap-junction-like intercellular connections could be involved in GADC formation in sea urchins.

### 3.2. Apical Surface Netrin as an Egression and CBAS Extension Signaling

The next consideration regarding the present egression mechanism was the molecular interaction that directs the process. Netrin on the larval body surface, around the exit of GADCs before the onset of egression, as shown in this study, may be signaling the blastocoelar GADCs to egress toward the larval body surface and align there. 

The present Netrin peptide sequence is a homologue of mouse Netrin-1, ARRCRFNMELYKLSGRKSGGVC sequence, that is known to be involved in the Netrin1/Unc-5 interaction [22]. The present synthetic CRFNMELYKLSGRKSGGVC peptide from Hp-Netrin severely inhibited GADC egression, and the following CBAS patterning process as well. This may be due to the peptide binding to the Netrin receptor immunoglobulin domain (Ig domain) of Unc-5, which seals the binding site of the complete form Netrin. This may cause an excessive repulsive reaction to GADCs by the blastocoelar Netrin, and thus cause egression inhibition. In the blastocoel, such an interaction may also inhibit the attachment of GADCs to the Netrin-coated spicules; again, as was seen in this study. Consistent with deprived CBAS formation, the ciliary beating activity of the ciliary band diminished accordingly. Our previous report on the inhibition of axon extension from the serotonergic apical ganglion and declined larval swimming activity was based on a different synthetic peptide, 181SQDFGKTW188, of Hp-Netrin1, that was derived from the far C-terminal side [14]. The present results showing that the serotonergic apical ganglion extended short cell processes toward the CBAS (Figure 2C,D) may imply the presence of a serotonergic/GABAergic crosstalk mechanism in the ciliary beating activity.

A role of Netrin as an axon guidance cue has been reported before [33,34,35,36,37,38]. In this process, Netrin interacts with the deleted in colorectal cancer (DCC) receptor, along with Unc-5 and co-factors for signaling. In the present sea urchin larvae, Unc-5-expressing CBAS was localized diagonally to the circumoral ectoderm side on the oral side beneath the Netrin stripe by the ciliary band, but not right under the Netrin stripe (Figure 5(E–F5)). This suggests Unc-5-expressing CBAS is not solely under navigation control by the Unc-5/Netrin interaction; rather it requires another Netrin receptor, such as DCC, that in contrast to Unc-5 attracts Netrin. However, although DCC homologs are reported in the present sea urchin database, HpBase [30], no functional studies of sea urchin DCC have been reported to date. Furthermore, since the Netrin molecule binds to multiple receptors other than DCC [39], such as the DCC/Neogenin complex, Down syndrome cell adhesion molecule (DSCAM), Netrin G ligands (NGL) [40,41], and DCC linked kinases, including focal adhesion kinase (FAK), non-catalytic region of tyrosine kinase 1 (NCK1), Src family kinase (SFK) [41], these also need to be explored in the future studies.

### 3.3. Netrin Stripe along the Ciliary Band

The present Netrin stripe along the ciliary band seems consistent with a previous in situ report on sea urchin larva by Slota et al. [21] that showed the Netrin gene expression site on the circumoral ectoderm side of the ciliary band. The Netrin gene expression site by the ciliary band is reminiscent of the midline ectoderm in vertebrates and invertebrates [42,43,44]. The circumoral ectoderm side of the ciliary band thus may play a similar role to midline ectoderms in Netrin expression. In addition to Netrin synthesis sites, it has been proposed that protein localization seems to be dependent on a substantial number of genes, such as unc-18 and unc-68 in *Caenorhabditis elegans* [45] and Frazzled/Unc-40 in *Drosophila melanogaster* [46]. Thus, future sea urchin Netrin studies will almost certainly unveil more molecular detail. 

## 4. Materials and Methods 

### 4.1. Larval Preparation 

Sea urchins, *H*. *pulcherrimus*, were collected in the vicinity of the Research Center for Marine Biology, Tohoku University, Japan, or the Marine and Coastal Research Center, Ochanomizu University, Japan. Gametes were obtained by intracoelomic injection of 0.5 M KCl. Eggs were inseminated and incubated in filtered seawater (FSW) and raised in 24-well plates at 30–40 embryos/2 mL well at 18 or 20 °C, until the 8aPL stage. Larvae were fed with around 10,000 cells/mL *Chaetoceros calcitrans* (Nisshin Marine Tech. Ltd., Yokohama, Japan), from four days after fertilization until the day that is described in the text.

### 4.2. Whole-Mount Immunohistochemistry 

The larvae that reached the developmental stages described in the text were fixed in 4% paraformaldehyde (diluted in FSW) for 15–20 min at ambient temperature (AmT) and dehydrated in a series of increasing concentrations of ethanol, starting from 30% (*v*/*v*), and stored in 70% ethanol at 4 °C until use. For WMIHC, the samples were hydrated in decreasing concentrations of ethanol to 30% and transferred to 0.1 M phosphate buffered saline with 1% (*v*/*v*) Tween-20 (PBST; Medicago AB, Uppsala, Sweden). After further washing in 0.1 M PBST, the samples were blocked with 1% (*w*/*v*) bovine serum albumin in PBST for 1 h, and exposed to primary antibodies (Abs) in PBST for 24 h at 4 °C in various combinations for multi-stained WMIHC (Table 1). These Abs were then washed in PBST three times (10 min each) and were visualized with secondary Abs that were diluted in PBST. The secondary antibodies used were Alexa Fluor 488- or 594-tagged goat anti-rabbit or mouse IgG Abs (Invitrogen, Carlsbad, CA. USA; diluted 1:200 to 1:500). They were incubated with the samples for 2 h at ambient temperature. Most of the samples were counterstained with 1–2 μg/mL 4′,6-diamidino-2-phenylindole (DAPI). To identify the ectodermal region, the 16-cell stage embryos were stained for the actin filaments with Rhodamine phalloidin and incubated until 4aPL according to the methods described previously [2]. Then, they were further stained with anti-Hp-GAD Ab to specify the CBAS region. They were examined under a Micro-Radiance confocal laser scanning microscope (CLSM; Bio-Rad Microscience, Hemel Hempstead, UK) or a TCS SP8 CLSM (Leica Microsystems, Co. Japan, Tokyo, Japan); otherwise, they were analyzed as described in the text. Images were analyzed with a public domain of ImageJ 1.52a (National Institutes of Health, USA; http://imagej.nih,gov/ij).

### 4.3. 3D Image Reconstruction

To clarify the spatial relationship of the images obtained with CLSM, the images were reconstructed three-dimensionally with the 3D visualization and analysis software Avizo ver.6.1.1 (FEI Visualization Sciences Group, Bordeaux, France) or Amira (FEI Visualization Sciences Group, Burlington, MA, USA).

### 4.4. Counting the Number of GADCs in Each CBAS of Larvae

The number of GADCs in each of the CBAS during pattern formation was counted under the fluorescence microscope using the larvae that were double-stained with anti-Hp-GAD ab and DAPI. 

### 4.5. Generation of GFP-Tagged Hp-Unc-5v2

Hp-Unc-5 was detected in 4aPL after GFP:Hp-Unc-5 microinjection of unfertilized eggs, as described below and according to our previous report [14]. Briefly, GFP-inserted pCS2+vector (pCS2+GFP) was generated by ligating the coding sequence of the tagGFP2-C GFP vector (Evrongen, Moscow, Russia) to pCS2+ vector. The coding sequence of Hp-Unc-5v2 was amplified by the following primer set from the cDNA template of 36-hpf prism larvae.

R-v2: 5′-TCACCGAATTCATGAGACGCCGAAGTGCCGG-3′
R-v2: 5′-ACTCGCTCGAGCYGTACATGGCCTTCAAACA-3′

The amplicons were cleaved with EcoR1 and Xho1 and ligated to pCS2+GFP. The vector was cleaved with Ncol, and capped mRNA was synthesized using a mMESSAGE mAMACHINE SP6Kit (Ambion, Austin, TX, USA). Then, 3 mg/mL of the synthetic mRNA was microinjected into unfertilized eggs. They were inseminated and raised in a culture dish in an incubator at 18 °C until 4aPL. The samples were fixed in 4% paraformaldehyde and processed for WMIHC using anti-Hp-GAD antibody, as described above.

### 4.6. Intervention for Proper Patterning of CBAS by the Unc-5 Binding Ig Domain Peptide of Hp-Netrin

The prism stage larvae were incubated at 15–20 larvae/mL in filtered plain sea water (Control) or with 5 μg/mL synthetic Hp-Netrin peptide in 30 mL plastic tissue culture flasks until the 3dpf-4aPL stage. The peptide CRFNMELYKLSGRKSGGVC (*Hemicentrotus pulcherrimus* Genome and Transcriptome 

Database: http://cell-innovation.nig.ac.jp/cgi-bin/Hpul_public/Hpul_annot_search_output.cgi) was synthesized by GenScript Hong Kong Inc. The sequence constitutes a part of the Unc-5 immunoglobulin domain binding site of Hp-Netrin [22]. 

### 4.7. Swirling Track Pattern Analysis

To obtain the swirling track pattern created by the ciliary beating, the larvae were transferred to whole grass slides with marine algae, *Chaeteceros gracilis* (Nisshin Marine Tech. Ltd., Yokohama, Japan) as a marker of swirling [22], and then dark-field photographed with a Canon EOS Kiss X3 camera with darkfield for 3.2 s of exposure time. The images were converted to black and white images and the contrast enhanced to visualize the swirl pattern better with ImageJ 1.52 (NIH). Twenty-two of the peptide-applied larvae and 35 of the peptide-free controls were examined. 

## Figures and Tables

**Table 1:** List of antibodies used in this study and their applications.

Primary Antibody	Applied Dilution	References
Hp-GAD rabbit	1:500	[14]
Epith-2 mAb mouse	1:100	[13]
5HT rabbit (S5545)	1:1000	Sigma-Aldrich, St. Louse, MO, USA
Hp-Netrin rabbit	1:50	[17]
Hp-Dopamine receptor D1 mouse	1:500	[18,19]
Hp-5HThpr mouse	1:200	[4,47]

## Supplementary Materials

Supplementary Materials can be found at https://www.mdpi.com/1422-0067/21/18/6587/s1.

## Abbreviations

GADC	Glutamate decarboxylase-expressing cell
dpf	Days post-fertilization
CBAS	Ciliary band associated strand
r-, l-ala	Right or left antero-lateral arm
r-, l-latc	Right or left lateral CBAS
poc	Post-oral CBAS
preoc	Preoral CBAS
CLSM	Confocal laser scanning microscopy
DRD-1	Dopamine decarboxylase D-1
WMIHC	Whole-mount immunohistochemistry
GFP:Unc-5	Green fluorescein tagged Unc-5
